# Early marriage, education and mental health: experiences of adolescent girls in Mozambique

**DOI:** 10.3389/fgwh.2024.1278934

**Published:** 2024-06-12

**Authors:** Joaquim M. Nhampoca, Jeanette E. Maritz

**Affiliations:** Department of Health Studies, University of South Africa, Pretoria, South Africa

**Keywords:** adolescent, girls, early marriage, education, mental health

## Abstract

**Introduction and background:**

Early marriage and maternity represent a violation of human rights and a barrier to achieving gender equality in education. Studies conducted across various regions and countries, including Mozambique, have highlighted the negative consequences of early marriage and maternity, particularly on adolescent girls' physical, educational and mental health. Adolescent girls who marry before the age of 18 are more prone to a broad spectrum of mood, anxiety, and other psychiatric disorders. In the districts of Maganja da Costa and Morrumbala in Zambézia Province, Mozambique, high rates of early marriage persist despite government policies and interventions.

**Purpose statement:**

This study aims to understand early marriage's socio-cultural, economic, and psychological drivers and impacts on adolescent girls' lives, focusing on education, mental health, and well-being.

**Design and methods:**

This paper used a qualitative research design. We employed a life-story approach and used purposeful sampling to identify participants. Semi-structured interviews were conducted with 25 participants and the data were analysed using Tesch's thematic analysis approach.

**Results:**

Elements contributing to early marriage and pregnancy are intricately linked with socio-cultural elements. These include the induction into specific societal roles, the affirmation of childbearing, the perceived importance of dowry, the impact of rite-of-passage ceremonies, and the cultural significance associated with a girl's initial menstruation. School dropout often originates from geographical and transportation challenges, nudging adolescent girls towards early marriage. In some instances, termination of pregnancy was viewed as a solution, while engaging in sexual activities was seen as a means to inject purpose into their lives or as a response to poverty. Positive changes and emotions were evident, yet regrettably, the ramifications of early pregnancy and marriage were primarily detrimental. These influenced the adolescent girls' interpersonal connections, educational and career opportunities, emotional well-being, and access to support networks.

**Conclusions:**

The modern perspective, which advocates for equal educational rights for boys and girls and posits that marriage should occur after the age of 18, contradicts the socio-cultural perspective of adulthood.

**Contribution:**

This study adds to the ongoing efforts to prevent and combat early marriage and maternity in Africa.

## 1 Introduction

Child marriage, transcending borders, cultures, ethnicities, and religions, infringes on human rights (1) and often impedes adolescent girls from reaching their educational and mental health potential. This practice, involving individuals under the age of 18 (2), frequently results in adolescent pregnancies, with extensive physical, educational, and mental health repercussions. The global commitment to eliminate child, early, and forced marriage by 2030, integral to the Sustainable Development Goals (SDGs) (3), is crucial; failing this target threatens progress across several SDGs, notably in education and mental health domains.

Research in India and West and Central Africa underscores child marriage's prevalence among uneducated, impoverished, and rural women, revealing that girls with secondary or higher education are less likely to marry before 18 (4, 6). Hence, enhancing girls' educational access and providing financial assistance to needy families are pivotal in eradicating child marriage.

Studies in Ethiopia, Indonesia, Kenya, Malawi, Mozambique, and Zambia identify poverty and economic conditions as primary child marriage drivers. Nevertheless, paternal education is a protective factor in Ethiopia, Kenya, and Mozambique (7). In Indonesia, child marriage is often deemed a preventive measure against premarital sex or pregnancy. The phenomenon is a reflection of societal norms and, in some instances, a result of girls' agentic choices, with pregnancy often precipitating marriage in regions like Malawi and Mozambique (8).

Mozambique, this study's focus, exhibits one of the highest child marriage rates globally, with nearly half its girls married before 18 and a significant portion before 15 (9). Despite its severity, Mozambique, like other sub-Saharan African nations, receives comparatively less attention than countries like India, Bangladesh, and Ethiopia (10).

A Mozambique study on school dropout causes suggests that disseminating and clarifying legislation through schools could mitigate child marriage by raising awareness among girls, including those married or at risk, alongside engaging parents and community leaders (11). Further findings associate school dropout with early pregnancy and related health issues in Mozambique's rural settings (12), paralleled by research in Nepal indicating a significant correlation between early marriage and adverse health outcomes during childbirth (13).

Another study in Mozambique's Nampula and Rapale districts highlights the influence of initiation ceremonies on teenage pregnancy and child marriage, underscoring the need to address violence and sexual abuse to empower youths in making informed sexual health decisions (14). Thus, enhancing comprehensive sex education access, especially for out-of-school youth, and addressing violence and sexual abuse within programs is imperative.

Preventing child marriage requires heightened awareness, law enforcement, and strengthened legal and policy frameworks (6). Sensitisation campaigns in rural areas and making educational institutions more accommodating for young married girls are essential strategies to deter child marriage and encourage girls' educational continuity (15).

Interventions must span individual, family, community, and national levels, incorporating civil and governmental efforts to enforce laws and raise awareness while advocating for gender equality and equitable educational opportunities for girls (6, 11, 13, 16).

The research gap in Mozambican studies on child marriage primarily lies in a nuanced understanding of the socio-cultural, economic, and psychological factors that contribute to the practice and its multifaceted impact on adolescent girls. Existing studies have yet to thoroughly explore the complex interplay of societal norms, economic pressures, and psychological effects that drive child marriage and influence the lives of young girls, particularly in terms of their education, mental health, and overall well-being. Additionally, there is a significant need for qualitative research that delves into the life experiences of girls affected by early marriage to capture their narratives, challenges, and coping strategies. Such insights are crucial for developing targeted, culturally sensitive interventions and policies to mitigate child marriage and support its victims.

This study aims to understand early marriage's socio-cultural, economic, and psychological drivers and impacts on girls' lives, focusing on education, mental health, and well-being. The objective was to explore and describe the life experiences of adolescent girls aged 10–19 who have encountered early marriage, maternity, or pregnancy.

### 1.1 Drivers of early marriage and maternity

Several key factors contribute to early marriage and maternity, including economic circumstances, socio-cultural practices, and the denial of educational access to girls, leading to unequal life opportunities.

Poverty is often cited as the primary catalyst for early marriage. Parents may marry off their young daughters to alleviate economic hardship by receiving a 'bride price' (17). Economic factors remain the primary drivers behind the persistence of early marriage (18).

In many African countries, including Mozambique, girls encounter significant cultural and social barriers that increase their risk of early marriage and maternity. Social pressure, cultural marital norms, religious prescriptions, and family shame resulting from unwanted pregnancy or premarital virginity loss can force girls into early marriages (19, 20).

In the context of Mozambique, socio-cultural practices related to early marriage often involve rites of passage or initiation, where adolescents are educated about societal life, including sexual health. These initiation rites can encourage early marriage, acting as a 'green card' for adolescents to begin their sexual lives, highlighting the power dynamics that place girls under male domination, limiting their freedom to choose their marriage partner and timing (21).

While economic and socio-cultural factors are the main drivers of early marriage and maternity, it's crucial to continue exploring other factors, as societal dynamics and social relationships can produce inequalities and power relations (22). The denial of girls' access to education and unequal life opportunities compared to boys can force girls into early marriage. Girls, particularly those recently out of school, are highly vulnerable to being married off, challenging the common assumption that school dropout precedes early marriage (23). Furthermore, societal tendencies often unfairly blame girls for early marriage and maternity, given their earlier sexual maturity than boys (1, 10).

### 1.2 Child marriage and mental health

SDG 3, “Good Health and Well-being” (3) aims to ensure healthy lives and promote well-being for all ages. This includes the promotion of mental health and well-being. This goal is particularly relevant for child-married girls as early marriage can lead to significant mental health challenges. Numerous studies, both pre- and post-COVID-19, have established a clear link between child marriage and the mental health of adolescent girls (24–27). A longitudinal cohort study from Uttar Pradesh and Bihar, India (28), found that poor mental health both preceded and resulted from child marriage. The study also revealed that early marriage increased the risks of depression and suicide. Abuse within the marriage could further heighten the risk of mental health problems and suicide in girls. A systematic review of 58 studies (10) indicated that five studies discussed the impact of child marriage on mental health or psychological well-being.

Girls who marry early may struggle with the responsibilities of childbearing and rearing, which could undermine their relational confidence (29). Additionally, the burden of household chores and social duties can be risk factors for depression. These responsibilities can lead to increased rates of depression, mood, and anxiety disorders. Child marriage can also be an isolating experience, further affecting the girls' psychological well-being (2, 30).

Intimate partner violence, poverty, childbirth challenges, and isolation were identified as causes of emotional distress in those married as children (2). Phobias, substance misuse, and antisocial disorders were reported less frequently. The authors warned that gaps remain in our understanding of child marriage and mental health issues. However, a study in Ghana suggested that child marriage seemed to provide some protection against measures of stress (31).

### 1.3 Child marriage and education

The Sustainable Development Goal (SDG) 4, “Quality Education” (3)^,^ is committed to ensuring inclusive and equitable quality education and promoting lifelong learning opportunities for all. However, child marriage, a prevalent issue particularly in regions such as sub-Saharan Africa, often disrupts girls' education, compelling them to leave school due to marriage, pregnancy, or domestic responsibilities (32, 33).

This complex interplay between child marriage, socio-cultural norms, and economic factors significantly impacts girls’ educational opportunities. For instance, research conducted in rural Gambia (32) revealed that child marriage negatively affects girls' education, leading to lower enrolment rates and challenges in transitioning to secondary or higher education. Economic constraints, such as poverty and socio-cultural practices, primarily drive these effects. Higher educational attainment is associated with improved health and longer lifespans, with tertiary education significantly influencing key health indicators such as infant mortality, life expectancy, child vaccination, and enrolment rates (34).

However, in some contexts, the link between marriage and education may not be as strong as generally believed. This could be due to substandard educational quality, resulting in inadequate skill development, leading girls and their parents to question the worth of girls' education (20).

## 2 Material and methods

This section highlights the design, sample procedures, data collection, analysis and ethical issues undertaken during the study.

### 2.1 Research design

This study used a qualitative research design (35). We employed a life-story approach (36) that focuses primarily on the symbolic life and meaning in individuals' lives while uncovering the patterns of social relations and the processes that shape them.

### 2.2 Sample selection

The study sample comprised 25 adolescent girls either living with a husband or parents at the time as a child married, adolescent mother or pregnant adolescent. The 25 adolescent girls were purposely (37) selected from secondary data of a cross-sectional survey previously conducted on the same targeted group by the first author in Maganja da Costa and Morrumbala districts in Zambézia Province. The selected adolescents were later invited to a life story interview in the current study.

The inclusion criteria included girls between the ages of 10 and 19, being early married, early pregnant and early maternity. The excluding criteria comprised undocumented girls who were not able to provide their birthdate, the age at which they became pregnant or had their first baby, and their current age, girls holding antenatal record cards with their age estimated by the maternal health service, not matching with the age presented by the interviewee (mainly when there was persistent doubt).

### 2.3 Data collection

The study used life stories and semi-structured interviews. Life story interviews provide a practical and holistic methodological approach for the sensitive collection of personal experiences that reveal how a specific human life is constructed and reconstructed in representing that life as a story (38). The interviews were done in Portuguese, Sena and Chuabo (the last two are local languages and were used for those participants who couldńt speak Portuguese). Therefore, before the interviews, we had to translate some terms into the local language (Sena), such as “early marriage” (Ku sembiua uzati fica thungha), “rite of initiation” (Ku vinirua) and “family planning” (Ku rera). This required hiring two native speakers of Sena and Chuabo (even though the first author could speak and understand Sena). The transcriptions were translated into English with accurate efforts to keep the meaning while considering cultural issues and perceptions. For instance, the use of the word “movement” to refer to child prostitution.

The first author (male) conducted semi-structured interviews consisting of open-ended questions. A trust relationship had already developed during a quantitative survey conducted in a similar study. Particular attention was given to ongoing rapport-building during the interviews. One way was to ask non-threatening questions first, such as educational level, the neighbourhood, and community affairs, before moving on to their childhood, dating, and relationships (39). The interviewer remained friendly, empathic and sensitive throughout the interview. Adolescents were asked if they were doing “okay” with the interview and where reluctance was observed, the interviewer paused the interview and only continued when the participant felt safe to engage further. The interviews took approximately 1 h to complete. The interviews were recorded using a digital recorder, with prior consent from the interviewee. Field and observation notes were also taken.

Being a male, interviewing females posed potential power and gender issues. Addressing power dynamics and the interviewer's gender in interviewing female adolescents was crucial for ethical and sensitive research. The male interviewer's awareness and adaptation of specific interview techniques, alongside appropriate gender sensitiveness, were pivotal in mitigating potential power imbalances and creating a safe space for the interviewees.

The inherent power differential between adults and adolescents, further compounded by gender dynamics, necessitates deliberate strategies to minimise its impact and foster an environment of trust and respect. The interviewer's approach, starting with non-threatening questions and being attuned to the interviewees' comfort levels, gradually built rapport and reduced the power distance. This strategy, alongside pausing the interview when necessary, emphasised the importance of consent and the adolescents' autonomy over their participation.

The interviewer's consistent display of empathy and sensitivity throughout the process was essential in maintaining a respectful interaction, which is particularly important given the sensitive nature of discussing personal experiences and the gender dynamics at play. The consent and assent were both written and then confirmed verbally during the interviews.

### 2.4 Data analysis

The collected data were analysed through open-thematic coding using Tesch's data analysis process (35). After transcribing the interview, the authors read the transcripts individually to understand the information and reflect on the overall meaning. The authors then started coding the data, organising it by categories and themes. While pondering potential meanings, tentative interpretations were made. Once this stage was completed, the authors held a consensus discussion to finalise the themes, categories, codes and the overall sense of the data.

### 2.5 Ethical considerations

The University of South Africa (UNISA), Health Studies Higher Degrees Committee (Nr. 483/2015) granted ethical clearance for the study. Thereafter, the National Committee of Bioethics for Health—Comité Nacional de Bioética para Saúde (Nr. IRB00002657) of the Ministry of Health—Mozambique, and authorisation from the Provincial Government of the site of study granted permission. Participation only occurred after the adolescent girls and their guardians were informed about the research and the content of the 'assent' or consent letter was explained. The adolescents' legal representative also signed the assent letter if required. Consent and assent were again confirmed verbally with each interview. To ensure the privacy of the participants and the integrity of their responses, family members, including parents and husbands, were informed about the potential negative impacts of their presence during the interviews. They were advised of the necessity for private communication between the researchers and the participants to facilitate open and honest dialogue. This measure was taken to prevent any undue influence or bias that might arise from the presence of family members. Additionally, all information about the participants was securely protected in a password-protected computer and was not shared with any third parties without a confidentiality agreement. To comply with the ethical issue of anonymity, the study used numbers to represent the participants instead of using their names.

## 3 Results

The discussion of the results is based on the themes, categories and codes from the interviews with the 25 participants. It resulted in four themes, as shown in Supplementary Table S2. Each theme, including the concomitant category and code, is discussed along with verbatim quotes to support the statements. The authors would like to mention one caveat. In these communities, the norm is that sexuality is sacred and not to be shared. Almost all the adolescent girls said that they “*forgot*” or became “*shameful*” (P 9, 11, 21 and 24) when the topic was raised. The results must thus be read within this context.

### 3.1 Understanding the dynamics leading to child marriage and adolescent pregnancy

To understand adolescent girls' life stories, one needs to understand the dynamics leading to their marriage and pregnancy. Several socio-cultural meanings were attached to understanding the dynamics of early marriage and adolescent pregnancy. These include socialisation into societal roles, legitimising children, the value and benefits of bridewealth, the initiation rites and the social meaning of the first menstruation. This could suggest the existence of multiple factors propelling adolescent girls to early marriage and childbearing.

#### 3.1.1 Socio-cultural meanings

In their interactions, people share meanings and make sense of their lives in a constructed world. In **socialising into societal roles**, girls are taught from childhood to assume the role of a housewife and take on domestic affairs such as washing clothes, dishes, cleaning, and cooking. Girls are also seen at an early age as future wives and mothers.

*“…for my case at least, I already know doing some works, I know how to do such things for man [husband], I know because when I lived with my mother I used not to sit only, doing nothing. That is why I know how to do something, wash clothes, and clean the house and if a man wants breakfast. My mother used to teach me and say that you will not stay with me forever, you must learn something”* [P8, 18 years, 2 years married, 1 child].

“*Wake up, clean the yard, clean the dishes, clean the house, fetch water, take bath and go to school”* [P24, 17 years, single, pregnant adolescent]. A significant disparity in the distribution of household chores between men and women was found in women shouldering a larger share of these tasks (40). This imbalance is further underscored by the perception of involvement, which is lower among men than women. Traditional gender ideologies play a pivotal role in shaping the division of household chores. Tasks typically perceived as feminine, such as washing, ironing, shopping, cooking, or cleaning, are predominantly undertaken by women. Furthermore, these gender ideologies imbue household chores with different meanings for men and women, further perpetuating the unequal distribution of domestic responsibilities (41).

Child marriage and childbearing can also be understood from the perspective of **legitimising having children**. Some adolescent girls mentioned that the decision to get married was motivated by the need for a child. At a certain age in the community, there is such an expectation because being a mother represents a certain status and having a companion—a child meant someone who could help their parents in domestic affairs. This was often part of the girls' life story as they were conceived for the same reason. Having a child was not seen as a negative consequence.

“*For me having a child is something good. I do not know for others…because you can ask* [the child] *to do something, play with him. The same they* [the girl's parents] did with me” [P16, 17 years, 2 years married, pregnant adolescent].

From a feminist perspective, adolescent pregnancy and childbearing can indeed be viewed positively. This perspective emphasises the potential of young mothers, recognising them as capable, loving, and nurturing individuals (42). As per this viewpoint, the real crisis is not the pregnancy or childbearing itself but the lack of adequate support systems for these young mothers (43). This lack of support can manifest in various forms, including financial, emotional, and societal, thereby creating challenges for these young women.

The **value and benefits of the bridewealth** refer to adolescent girls being subject to child marriage whereby their parents get the benefits of the bridewealth (44). This includes money, property, or other wealth given by the groom or his family to the bride's parents or family. The bridewealth is a kind of legitimisation of the marital relationship between a girl and a man/boy. For some parents, it means a reward and compliance with social norms:

“*Here it is like that when a man brings money they say yah; the man is that one you have to marry him”* [P8, 18 years, 2 years married, 1 child].

“…*he went to my house for lobolo* [bride ceremony], *paid some money, gave something* [clothes, shoes, brewery and others] *and then took me* [as his wife]” [P14, 17 years, 3 years married, 1 child].

Bridewealth could be seen not only as a legitimisation of the marital relationship between a girl and a man/boy or as compliance with social norms but also as a strategy used by parents for their subsistence by receiving financial and material resources from their son-in-law, a paying back the investment they made in their daughters. A study found that as the practice of bridewealth marriage diminishes, its significance has transformed. It now represents personal wealth and prestige more than a symbol of familial authority (44).

The **initiation rites** relate to different discussions and approaches regarding initiation, focusing on sexual and reproductive health, violence against girls, and school dropout. However, these perspectives differ from the community perspective based on the function of the initiation rites. The initiation rites are just one embedded factor in the network of social relations influencing child marriage. Many participants in the study were aligned in their responses. They considered the rites of initiation to social integration and becoming a full member of society by learning or acquiring different roles, manners and practices:

“*We have to respect our husbands, to know how to take care of them, not be greedy, participate in death ceremony, take care of the piece of clothes we use during menstruation, dressing. This kind of things which are said to be a woman.”* [P9, 17 years, single, 1 child].

*“When you leave [after the rites of initiation ceremony] you have to respect your mother and father. Early in the m*orning, you have to salute them, clean the house. Whatever your father says you have to obey” [P7, 18 years, 4 years married, 1 child].

According to a participant, the education that girls received in the rites of initiation put them in a position of submission to men:

“(smile)…do whatever your husband wants, put water in the bathroom for him, cook for him and set the table” [P21, 19 years, 3 years married, 1 child].

This literal submission should be understood as part of the social roles that girls and boys are taught, not only during the rites of initiation but also from the education they receive from their parents. Their actions are embedded in networks of social interactions and shaped by society. Participating in the initiation rites represented the girl being allowed to enter the world of adults and become a woman. If a girl did not participate in the rites of initiation, she was likely to be excluded or not acknowledged by other girls or women:

*“…because before I went to rites of initiation, my friends [peers] used to laugh at me saying that I was ‘nothing’. It left me upset”* [P15 17 years, 3 years married, 1 child].

The initiation rites aim to respond to a social function (45). They are mandatory for boys and girls of a certain age, representing a transition into a new world of adults. Those who are initiated are taught about the future when they are grown up, how they should act, what to do, how to behave and acquire social norms and values common to society. Therefore, initiation rites are considered socially useful because they contribute to the social cohesion and integration of members of a certain group.

Some interviewed child-married, adolescent mothers and pregnant adolescents recognised that they were still a child (considering their age). Still, because of the **social meaning of the first menstruation**, they found themselves in an ambivalent situation. Traditionally, they were seen as women, ready to be wives and mothers:

*“…Since you get the first menstruation at 12 years, that's all. That is the reason why the majority when is back home [from the rites of initiation] think about falling in love and marrying”* [P9, 17 years, single, 1 child].

The first menstruation is also a starting point for learning about sexuality, marriage, and hygiene practices. This means a transition to adulthood and has some practical consequences:

“(shy smile)… *they told me about what to do during menstruation, hygiene* [bath], *husband*” [P14, 17 years, 3 years married, 1 child].

The onset of menarche, marking the beginning of puberty and reproductive maturity in girls, is a significant developmental milestone (46). This experience and subsequent menstruation are deeply rooted in socio-cultural norms and practices, influencing how women manage their menstrual health with dignity. Studies further highlight the repercussions of negative menstrual experiences, which can extend beyond physical and psychological health (47). These experiences can also impact various aspects of a woman's life, including her education, employment, and social participation.

### 3.2 Education matters

This theme explores the intricate relationship between family income, geographical distance and access in the form of transport and school dropout rates, with a particular focus on how these factors contribute to early marriage. The scarcity of resources necessary to complete schooling often pushes adolescent girls into early marriage, highlighting the critical role of economic factors in educational attainment and marital decisions.

#### 3.2.1 Family income, geographic and transport issues

The adolescent girls shared their challenges. The **lack of resources** to complete schooling due to the family's low income was concurrent with school dropout. Some child-married girls pointed out:

*“I was going to school, but because of a lack of resources, I did not continue, and then decided for marriage”* [P6, 18 years, 2 years married, 1 child].

*“What to do! I have no parents, even anyone to help me. I live with my grandparents”* [P3, 16 years, 2 years married, 1 child].

UNESCO (48) asserts that millions of learners risk leaving school prematurely due to financial difficulties, pressure to find employment, household responsibilities, early or forced marriage, and/or unexpected pregnancy. These factors often result in a diminished interest in education among these learners. Additionally, household income is a significant risk factor strongly correlated with school dropout rates (49).

Poverty plays a pivotal role in decisions and practices concerning early marriage, especially in low-income societies compared to wealthier ones. The lack of resources in these societies hinders the provision of healthier alternatives for girls, such as extended education and skills training, which could guarantee a more secure future (50).

The lack of resources to complete schooling is further exacerbated by geographical and transport challenges, contributing to school dropouts.

*“To continue with my studies, I have to shift from my community which is 30–40 km away, to do grade 8. Some girls got support from their parents buying a bicycle, but it may also not be safe”* [P1, 19 years, single, 1 child].

According to UNESCO (48), the geographical location of schools can significantly impact children's ability to attend. For some families, the cost or availability of transportation may be prohibitive, making schools too distant for safe travel. Furthermore, infrastructure issues can negatively affect school attendance and educational outcomes, whether inaccessible or unsuitable.

**Limited opportunities often result in menial jobs**. Adolescent girls, particularly those from underprivileged families or with scarce resources, are frequently dispatched to cities or main villages for menial work. Alternatively, they may be compelled to undertake such tasks to provide for their child.

*“In Quelimane [the administrative capital of the Zambezia Province] it was also like that. They took me to take care of her daughter”* [P17, 17 years, 2 years married, 1 child].

*“If I had some other thing to do because in the agriculture field [where she helps her mother to grow crops] we only get food, and the child needs clothes and many other things”* [P25, 18 years, single, 1 child].

Studies have found that across Africa, tens of thousands of children share a common, unfortunate fate—they are forced to work instead of pursuing an education (21, 32). This predicament deprives children of a promising future and excludes them from potential social and economic advantages. The issue is particularly prevalent in rural areas, where child labour often supersedes educational pursuits, leading to a cycle of poverty and limited opportunities.

### 3.3 Mental health and emotional states

Various emotional states resulted from child marriage and childbearing, including emotional distress such as regrets and challenging interpersonal relations.

#### 3.3.1 Emotional distress

Participants in this study often voiced their feelings of **regret and feeling trapped** over decisions they made or were forced to make and the potential impact on their education. In addition, the phrase "*What can I do*?" below may suggest feelings of **helplessness** and a perceived lack of options.

*“Do you think that I have any idea? I have no idea. If you are married, there is nothing else. What can I do?… If I had continued with my studies, it would be better. I am suffering… I often say, why did I get married”* [P6, 18 years, 2 years married, 1 child].

The adolescent below might demonstrate underlying emotions of **guilt or shame** given that the adolescent girl refers to the early pregnancy as a “*mistake*”.

*“I committed a mistake by becoming pregnant earlier”* (P2).

In addition to regret, the participant below may demonstrate feelings of **stagnation or frustration**.

“*If I knew I wouldn't be pregnant. I regret that because my daughter was not accepted by her father. Now I am still at home living with my mother…”* [P10, 18 years, single, 1 child].

Early marriage often results in regret and a profound sense of missed opportunities (49). This finding is further corroborated by a report from Human Rights Watch (51), which conducted interviews with numerous girls who had experienced early marriage. These girls expressed significant emotional and psychological distress due to their early marital experiences. They reported widespread dissatisfaction with their marriages and harboured deep regrets about their early marriages. When faced with the multifaceted responsibilities of motherhood, numerous adolescents often feel constrained, as if in a metaphorical prison, unable to pursue their personal desires. This experience elicits a range of intense emotional responses, including fear and worry, regret and frustration, guilt and shame, and even depression (52).

#### 3.3.2 Interpersonal relations and emotional situations

The onset of pregnancy or marriage for a girl often triggers a shift in her social relationships. **Poor marital relations** can progressively deteriorate, particularly if the girl becomes a single mother or if she is in an abusive or **violent** relationship, leading to strained marital or social ties. Such situations can arise when the girl's parents do not approve of the pregnancy or the marriage in the first place.

“*I am not willing to live with the father of my child because he hurt me. When I was pregnant, he used to run away from me, but when he knew that I had given birth, his parents came to my home. I was about to kick them, but my grandmother said not to do that because they were people”* [P2, 17 years, single, 1 child].

Some parents forced their daughters to live with the man responsible for their pregnancy:

“*When I was pregnant …My parents were nearly to kick me away from home. They determined a limited time so that I could go. Then, they reconsidered and said to stay home”* [P25, 18 years, single, 1 child].

Mozambique had a prevalence of 17.5% for girl-child marriage and intimate partner violence. Young women who began cohabiting or married before 18 were more likely to experience violence (36.9%) than those who did so at 18 or older (32.5%) (53). This pattern was consistent across physical, emotional, and sexual violence. Even after accounting for socio-demographic factors, child marriage was more strongly associated with intimate partner violence than adult marriage. According to UNICEF (2), girls who marry before the age of 18 are more likely to experience domestic violence and less likely to stay in school. Women who experienced child marriage were more prone to marital disruption and less likely to report domestic violence (54). During times of civil unrest and natural disasters such as the Ebola virus and COVID-19, various forms of violence, including sexual, physical, and emotional exploitation, early and forced marriages, child trafficking, and child labour, could escalate (55). This is particularly alarming in a country where girls' education is already challenging.

Some girls were forced into marriage to avoid “*People's pregnancy*” (a pregnancy that occurs outside of marriage, particularly by an unknown male). This situation is seen as dishonourable and shameful for the girl's family within the community. To avoid this, families sometimes accept child marriage as a preferable alternative.

*“My mother told me, now you are grown up. It is your fourth year after the first menstruation. You can happen to be pregnant of anyone, people's pregnancy”* [P23, 18 years, 3 years married, 1 child].

Additionally, girls who are victims of domestic violence often have no alternative to marrying early (or engaging in sexual life) in an attempt to flee from the suffering. This was more common among girls not living with their parents, such as orphans and vulnerable children. This could potentially strain family and peer relations and impact the adolescents' self-esteem:

“*When I passed to grade 8* [in Quelimane city], *I said that I was going to spend the school holidays. When I arrived here* [Maganja da Costa] *I didn't go back because my aunt used to beat me…we were two girls, her daughter and I. When I was in the kitchen cooking she used to tell her daughter not to cook even do anything. I did everything alone. When I refused to do something she used to beat me”* [P16, 17 years, 2 years married, pregnant adolescent].

Violence against girls was also prevalent among girls living with stepfathers. These girls found that marriage leads to freedom:

*“I lived with my stepfather. There was always a shout at home with my mother. Always shouting, I, eh, I can't support these things…that is the reason why I decided to marry”* [P17, 17 years, 2 years married, 1 child].

Child marriage and childbearing are also an issue of gender. Girls, differently from boys, are subject to the maternal role and are often voiceless and powerless (56).

### 3.4 Making choices

The precarious reality of adolescent girls leads to making decisions on a distressingly complex dimension. Often grappling with emotional distress, these young girls are confronted with crucial choices that extend to abortion as a remedy, engagement in sexual practices as a means to make a living or survive, but also in positive emotional responses. Some girls had no choice but to resign themselves to their fate or to conform.

#### 3.4.1 Abortion as remedy

The parents' reactions or the experiences of other girls/family members may cause some pregnant adolescents to consider abortion. The intention to abort was commonly invoked as a potential remedy:

*“… I just wanted to abort. I was afraid of my mother. I said if my mother gets to know that I am pregnant what will happen? That is why I wanted to commit an abortion. But when she found out that I was pregnant she did not insult me or hurt me”* [P8, 18 years, 2 years married, 1 child].

*“…when I was sixteen years I became pregnant. I decided for an abortion but it was unsuccessful”* [P22, 19 years, single, 1 child].

Economically dependent adolescents stand to lose more if others disagree with their decision to abort. Disapproval could lead to the threat of revealing the pregnancy and abortion decision to their parents, potentially preventing the procedure. Fearing judgment, adolescent girls often keep their decisions private despite yearning for emotional and material support (57).

#### 3.4.2 Engaging in sexual practices

The **scarcity of resources** was identified as a driving factor prompting engagement in sexual activities. Engaging in such practices was discovered to be a means of earning a living.

The issue of poverty was recurrent in the interviews with parents and families of the interviewed adolescents. Poverty was understood by the participants as a lack of money to guarantee their subsistence, to satisfy their needs, a lack of school uniforms and materials, or a lack of someone to take care of a child.

*“…now things are worse because of poverty. At the beginning of the night in the market, you find a crowd of children. I ask myself ‘What will I eat, wear, and perform the hair? So where will I get these? The young boys can*'t give this to me.” [P18, 17 years, single, pregnant adolescent].

Transactional sex, unlike sex work, is characterised by non-marital, non-commercial sexual relationships driven by an unspoken agreement that sex will be reciprocated with material support or other benefits (58). Transactional sex is more widespread than sex work. In African countries, the prevalence of transactional sex among adolescent girls and young women varies extensively, with estimates ranging from a conservative 2.1% to a significant 52% (58). Factors such as psychological well-being, skill sets, and cognitive abilities can significantly impact youth engagement in sexual activity (59). Emotional distress, symptoms of depression, negative self-perception pose and goal-directed sexual engagement often lead to the initiation of sexual intercourse. These insights continue to underscore the multifaceted nature of motivations that guide sexual behaviour, encompassing material needs, societal status, and personal psychological and emotional states.

#### 3.4.3 Positive emotional responses

Early maternity is sometimes linked to positive emotional responses in that “*a child* [can]*bring happiness”* (P3). Some adolescent girls reported a mixture of love, determination, and perhaps a sense of responsibility or duty to care for their child.

*“I do not regret becoming a mother. I love my daughter a lot. That's it. I will give her the best future, do everything good for her”* [P1, 19 years, single, 1 child].

The adolescent girl below displayed a strong conviction to live her life on her terms, even if that meant leaving her family.

*“I said I am going away because my dad didn't want me to have a child. Then, I said I am leaving your house so that I can live freely and have my child”* [P21, 19 years, 3 years married, 1 child].

The experience of pregnancy and parenting often prompts a transformative shift, sparking a positive reorientation. Some adolescents express a compelling desire for their children to seize opportunities and succeed in life (60).

#### 3.4.4 Complex emotional states

Some adolescents relayed a complex emotional state that suggests a struggle between **regret** for past actions, **acceptance** of current circumstances and **resignation or conformity**. They reflected on their childhood and what was happening to them.

*“…I took my own decision and fell where I was not supposed to. I did not listen to anyone's advice. Now, I am like that…I can only sell things as I was doing with my mother. Maybe doing that I can get some money to buy things for my child and me”* [P8, 18 years, 2 years married, 1 child].

The complex interplay of emotional states, encompassing feelings of regret, acceptance, and determination, highlights the considerable impact societal norms and pressures exert on individual decisions and their subsequent outcomes. These norms significantly shape and potentially limit the opportunities perceived by adolescent girls, thus influencing their responses to the situations they encounter (56).

Moreover, if a girl's mother conformed to these norms, such behaviour could be seen as the default or an essential route for these young girls within their community. This brings our discussion full circle, emphasising the pivotal role that socio-cultural interpretations and meanings play within adolescent girls' life stories.

## 4 Discussion

According to the results of this study, multiple factors propel adolescent girls to early marriage and childbearing, among them socio-cultural and economic factors. Traditional gender ideologies linked to the process of socialisation play a pivotal role. Girls are seen at an early age as future wives and mothers. Therefore, bridewealth legitimises the marital relationship between a girl and a man/boy, and it is a strategy used by parents for their subsistence by receiving financial and material resources from their son-in-law, which aids their subsistence. This practice perpetuates early marriage and positions it as a socio-economic transaction beneficial to the girls' family. These drivers of early marriage and childbearing align with other studies (7, 8).

Initiation rites are considered socially useful because they contribute to the social cohesion and integration of members of a particular group. If a girl did not participate in the rites of initiation, she was likely to be excluded or not acknowledged by other girls or women, further marginalising girls and limiting their social and educational opportunities. Regarding education, the lack of resources to complete schooling due to the family's low income and infrastructure issues, whether inaccessible or unsuitable, can negatively affect school attendance and educational outcomes, resulting in school dropout, often pushing adolescent girls into early marriage. Other studies pointed out the persistence of child marriage among uneducated, poor and rural women (4–6, 49, 50). suggesting a strong link between socio-economic disadvantages and the prevalence of early marriage.

Avoiding a pregnancy outside of marriage, particularly by an unknown male, led some adolescent girls to child marriage. In the studied communities, when a girl comes home pregnant, it represents dishonour and shame for the family. In some situations, the girls were willing to marry and become mothers as a means to avoid dishonour and shame for the family. This reflects both sociocultural and economic pressure as the family seeks to maintain its social standing through the institution of marriage. This was also suggested in studies in Indonesia and Mozambique (7, 8). As other studies have demonstrated, child marriage and childbearing are both associated with health concerns (7, 29).

## 5 Conclusion

The issues contributing to early pregnancy and marriage are entwined with socio-cultural aspects, such as the socialisation into specific roles, the validation of childbirth, the perceived value of bridewealth, the influence of initiation ceremonies, and the societal implications of a girl's first menstruation. The concerns of school dropout primarily stemmed from geographical barriers and transport difficulties, which consequently pushed adolescent girls towards early marriage. In some cases, abortion was considered as a remedy, and engaging in sexual practices was seen as a way to fill their lives meaningfully or as a response to poverty. Positive transformation and emotions were present. Unfortunately, the repercussions of early pregnancy and marriage were predominantly adverse, influencing physical well-being, interpersonal relationships, educational and career prospects, emotional health, and access to social support systems.

The interplay between child marriage, education, and mental health outcomes is complex and multifaceted, highlighting the need for comprehensive and context-specific interventions to address these interconnected issues. All programmes targeting child and adolescent marriage must integrate mental health and education with other strategies. The broader dynamic and context of early marriage and inequitable gender norms must be considered when advancing strategies to curb child marriage. A transformative paradigm could be useful in engaging with inequalities in terms of access to education, sexual and reproductive health rights, and health facilities. Therefore, building consciousness for girls' empowerment is critical.

This study focused on child marriage in a specific context and geographic area. Only girls were interviewed; boys could also be affected. Future research could investigate effective intervention strategies to counter the issue of early marriage and pregnancy. This could involve developing and testing educational programs to provide adolescent girls with knowledge and skills to make informed decisions about their lives, especially when they face economic pressures.

## Data Availability

The original contributions presented in the study are included in the article/**Supplementary Material**, further inquiries can be directed to the corresponding author.

## Supplementary material

Supplementary Table S1 Summary of qualitative sample.

Supplementary Table S2 Themes, categories and codes.
