# Supplementary figures and images for: Early marriage, education and mental health: experiences of adolescent girls in Mozambique
Source: Front Glob Womens Health. 2024 Jun 12;5:1278934. doi: 10.3389/fgwh.2024.1278934 (PMC11199522; doi:10.3389/fgwh.2024.1278934)

Supp 1


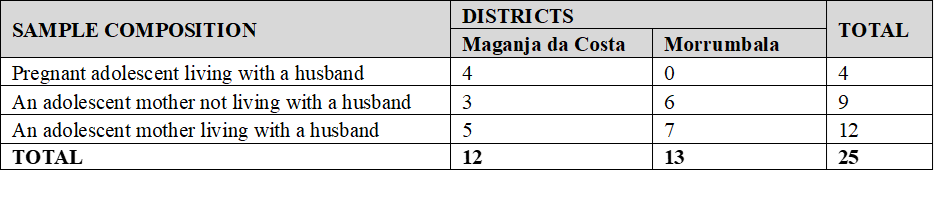

Supplement: Supplementary Table S1 — Summary of qualitative sample. [file Table1.docx]
