# Supplementary material for: Early marriage, education and mental health: experiences of adolescent girls in Mozambique
Source: Front Glob Womens Health. 2024 Jun 12;5:1278934. doi: 10.3389/fgwh.2024.1278934 (PMC11199522; doi:10.3389/fgwh.2024.1278934)
Supplement: Supplementary Table S2 — Themes, categories and codes. [file Table2.docx]

Supplementary file 2

Table 2: Themes, categories and codes

| **Theme** | **Category** | **Codes** |
| --- | --- | --- |
| 3.1 Understanding the dynamics leading to early marriage and adolescent pregnancy | 3.1.1Socio-cultural meanings | Socialisation into societal roles |
|  |  | Legitimising having children |
|  |  | Value and benefits of the bridewealth |
|  |  | The rites of initiation |
|  |  | The social meaning of the first menstruation |
| 3.2 Educational matters | 3.2.1 Family income, geographic and transport issues | Lack of resources |
|  | 3.2.2 Lack of opportunities leads to menial work |  |
| 3.3 Mental health and emotional states | 3.3.1 Emotional distress | Regret, feeling trapped, helpless, guilt, shame, stagnation and frustration |
|  | 3.3.2 Interpersonal relations and emotional situations | Poor marital relations and violence |
|  |  | ‘Peoples pregnancy’ |
| 4.1 Making choices | 4.1.1 Abortion as a remedy |  |
|  | 4.1.2 Engaging in sexual practices | Scarcity of resources |
|  | 4.1.3 Positive emotional responses | Happiness, love, determination, sense of responsibility |
|  | 4.1.4 Complex emotional states | Regret, acceptance, resignation and conformity |
